# Novel micelle PCR-based method for accurate, sensitive and quantitative microbiota profiling

**DOI:** 10.1038/srep45536

**Published:** 2017-04-05

**Authors:** Stefan A. Boers, John P. Hays, Ruud Jansen

**Affiliations:** 1 Department of Medical Microbiology and Infectious Diseases, Erasmus University Medical Centre Rotterdam (Erasmus MC), Rotterdam, The Netherlands; 2 Department of Molecular Biology, Regional Laboratory of Public Health Kennemerland, Haarlem, The Netherlands

## Abstract

In the last decade, many researchers have embraced 16S rRNA gene sequencing techniques, which has led to a wealth of publications and documented differences in the composition of microbial communities derived from many different ecosystems. However, comparison between different microbiota studies is currently very difficult due to the lack of a standardized 16S rRNA gene sequencing protocol. Here we report on a novel approach employing micelle PCR (micPCR) in combination with an internal calibrator that allows for standardization of microbiota profiles via their absolute abundances. The addition of an internal calibrator allows the researcher to express the resulting operational taxonomic units (OTUs) as a measure of 16S rRNA gene copies by correcting the number of sequences of each individual OTU in a sample for efficiency differences in the NGS process. Additionally, accurate quantification of OTUs obtained from negative extraction control samples allows for the subtraction of contaminating bacterial DNA derived from the laboratory environment or chemicals/reagents used. Using equimolar synthetic microbial community samples and low biomass clinical samples, we demonstrate that the calibrated micPCR/NGS methodology possess a much higher precision and a lower limit of detection compared with traditional PCR/NGS, resulting in more accurate microbiota profiles suitable for multi-study comparison.

The number of microbiota studies has rapidly increased since the introduction of next-generation sequencing (NGS) technologies and opened numerous new fields of research. This research often studies microbiotal changes as a result of various kinds of interventions and focusses on changes in the proportional microbial composition rather than actual microbial quantities. However, obtaining accurate and quantitative microbiota profiles makes high demands on the analytical sensitivity, specificity and reproducibility of standard NGS methodologies and requires a careful consideration of potential biases and bacterial DNA contaminations that can be introduced during the many steps of sample processing and sequencing^1^,^2^.

Recently, the authors published a micelle PCR/NGS (micPCR/NGS) methodology that limits both chimera formation and PCR competition, thereby reducing the introduction of PCR amplification biases into microbiota profiles^3^. However, 16S rRNA gene sequencing techniques still remain semi-quantitative methods, where the results are presented as proportional abundances of operational taxonomic units (OTUs), rather than absolute abundances of OTUs. This restriction lowers the reproducibility of microbiota profiling between different laboratories and does not reveal differences in absolute abundances of specific OTUs. For example, Hiergeist *et al*. observed high inter-laboratory deviations from an external quality assessment of 16S rRNA gene sequencing methods and concluded that there is an urgent need to develop microbiota profiling methods with an increased cross-study comparability^4^. This is of particular importance for the increasing implementation of 16S rRNA gene sequencing methods in the field of medical diagnostics, which requires high quality demands on the methods used. Further, the validity of 16S rRNA gene sequencing results are threatened by the presence of contaminating bacterial DNA derived from the laboratory environment and the consumables used in the experimental set-up^5^. Such contamination is particularly relevant for an accurate analysis of microbiota composition of low biomass samples (e.g. skin swabs)^6^,^7^. These contaminating DNA molecules can be derived from two sources: 1) contaminating bacterial DNA present within the sample-processing environment, and 2) contaminating bacterial DNA already present in the reagents/consumables used during sample processing. The introduction of contaminating bacterial DNA derived from the processing environment will occur randomly within the samples being processed and can be recognized by their non-reproducibility in multiple determinations of the microbiota of a particular sample. In contrast, the variety of contaminating bacterial DNA from reagents/consumables will be dependent on the manufacturer and batch numbers of the actual reagents/consumables used and will be present in all samples processed using these particular reagents/consumables.

In this report, we present a novel approach that employs micPCR/NGS in combination with an internal calibrator (IC) to determine the composition and absolute quantity of microbiotal genera. The IC used for this study consisted of quantified genomic DNA from a *Synechococcus* bacterium species that is absent in the natural microbial flora of the samples under investigation and allows the researcher to express the resulting OTUs as a measure of 16S rRNA gene copies by the use of a correction factor (sample OTU copies = sample OTU reads x (initial IC copies/IC OTU reads)). We utilized this calibrated micPCR/NGS approach to process a range of samples in triplicate in order to increase accuracy and to correct for contaminating bacterial DNA derived from the laboratory environment. In addition, contaminating bacterial DNA derived from the reagents/consumables used during micPCR/NGS were subtracted from samples via the use of a negative extraction control (NEC). To validate the calibrated micPCR/NGS approach (including the two-step strategy for removing contaminating bacterial DNA - as described above), we used a series of dilutions of an equimolar synthetic microbial community (SMC) sample and compared the results obtained against traditional PCR/NGS. Additionally, we evaluated the performance of our method to generate accurate quantitative microbiota profiles from actual low biomass clinical samples.

## Results

**Figure 1:**
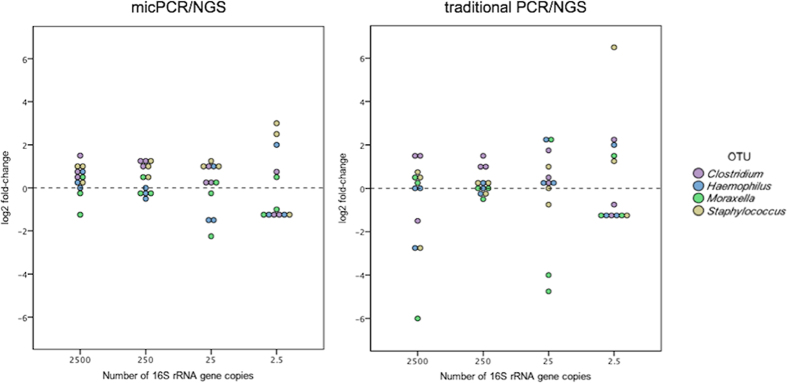
Precision of 16S rRNA gene copy determination using synthetic microbial community samples comparing the results of micPCR/NGS to traditional PCR/NGS. The synthetic microbial community (SMC) samples tested contained equimolar 16S rRNA gene copy numbers derived from four different bacterial species and ranged from 2,500 to 2.5 16S rRNA gene copies per species. Colored data points represent the individual measurements per bacterial OTU from triplicate experiments, corrected for the number of expected 16S rRNA gene copies and plotted using a binary logarithmic scale.

In order to determine the accuracy (trueness and precision) of the calibrated micPCR/NGS methodology, we utilized a 10-fold dilution series of a SMC sample containing equimolar 16S rRNA gene copies of *Clostridium perfringens, Staphylococcus aureus, Haemophilus influenzae*, and *Moraxella catarrhalis* (ranging from 2.5 to 2,500 16S rRNA gene copies per (mic)PCR of each bacterial species). Prior to amplification, *Synechococcus* DNA was added in such a concentration that each SMC sample contained 10% of IC 16S rRNA gene copies with a minimum of 50 copies for samples that contained less than 500 16S rRNA gene copies. The 16S rRNA gene V3-V4 regions were amplified in triplicate, using the same SMC/IC sample for each replicate, and sequenced using both micPCR/NGS and traditional PCR/NGS (as comparator). As shown in Supplementary Table 1, we obtained an average of 4,201 (±2,398) QC-passed sequence reads per SMC sample using both methods, of which the percentage of chimeric sequences was much lower for micPCR/NGS compared to traditional PCR/NGS (0.01% ± 0.03% vs. 4.56% ± 2.97%). Further, Table 1 shows the results obtained from triplicate experiments and indicates the accuracy for both methodologies used. The micPCR/NGS data, as well as the traditional PCR/NGS data, showed a median value of only a 1.3-fold difference between the measured 16S rRNA gene copies (average of triplicate experiments) and the expected 16S rRNA gene copies. Although these data suggest a similar and good trueness among both methods, the dispersal of replicate results varied greatly using traditional PCR/NGS. As shown in Fig. 1, the dispersal of replicate results obtained using micPCR/NGS was much smaller than using traditional PCR/NGS, indicating the higher precision of the micPCR/NGS methodology. For example, traditional PCR/NGS resulted in a coefficient of variation of 0.8, 0.5, 0.9 and 3.0 for the SMC samples containing 2,500, 250, 25 and 2.5 16S rRNA gene copies per bacterial species, respectively. In contrast, identical experiments using micPCR/NGS showed a coefficient of variation of only 0.4, 0.4, 0.6 and 1.4, respectively. The higher precision of the micPCR/NGS methodology lowers the number of random errors within 16S rRNA gene measurements and increases the repeatability of microbiota profiling results (Paired Wilcoxon signed-rank test; p < 0.01). As expected, the accuracy for both methods decreases as the number of template DNA molecules decreased due to the limited chance of successfully generating 16S rRNA gene amplicons at very low starting concentrations of DNA.

**Figure 2:**
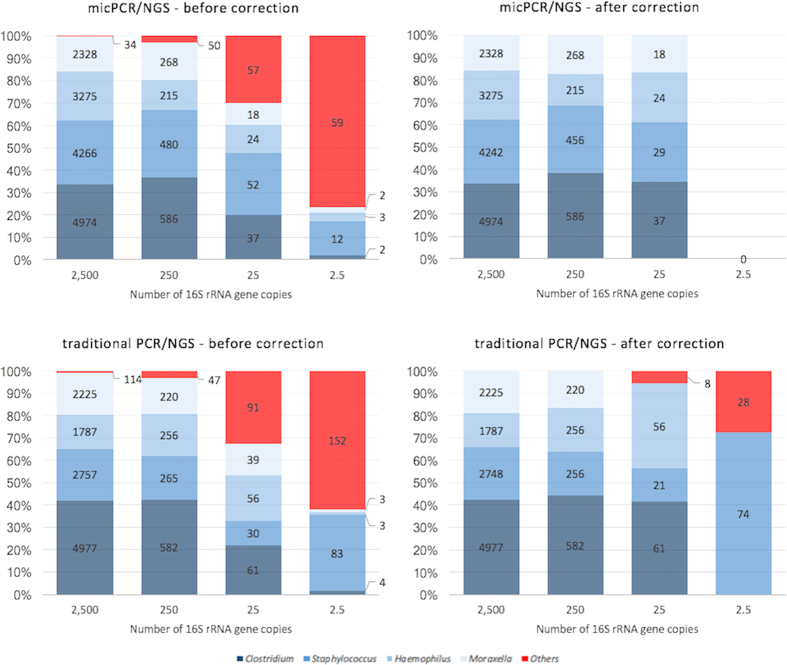
16S rRNA gene microbiota profiles obtained from synthetic microbial community samples comparing the results of micPCR/NGS to traditional PCR/NGS before and after correction for contaminating bacterial DNA. The synthetic microbial community (SMC) samples tested comprised equimolar 16S rRNA gene copies derived from *C. perfringens, S. aureus, H. influenzae,* and *M. catarrhalis* and ranging from 2,500 to 2.5 16S rRNA gene copies per bacterial species. Averages of triplicate micPCR/NGS and triplicate traditional PCR/NGS results are shown in 100% stacked bars before and after correction for contaminating bacterial DNA. The correction of contaminating bacterial DNA comprises two steps: 1) eliminating OTUs that could not be reproducibly measured in triplicate experiments, and 2) subtracting 16S rRNA gene copies that were also quantified in triplicate measurements of a negative extraction control (NEC) sample. Values within bars represent the calculated number of 16S rRNA gene copies per bacterial OTU.

Microbiota analysis is prone to the introduction of contaminating bacterial DNA molecules during sample processing. This is illustrated by the finding of 114 distinct OTUs that are represented by at least two or more sequence reads in our SMC experiments (Supplementary Tables 2–5). As expected, the number of distinct OTUs was maximal in the samples containing low amounts of input DNA, or no input DNA (NEC), leading to an unintended overestimation of microbial diversity. To correct for random bacterial DNA contamination from the laboratory environment, we processed each sample in triplicate and removed all OTUs that were not present in all of the three datasets obtained. To correct for bacterial DNA contamination from the reagents/consumables used, we subtracted the contribution of the NEC from each sample. The bacterial DNA contamination from reagents/consumables was calculated as the mean plus three standard deviations of 16S rRNA gene copies per OTU that were present in all three independent NEC measurements (Supplementary Tables 6 and 7). The quantified microbiota profiles obtained using micPCR/NGS and traditional PCR/NGS, before and after the correction of contaminating bacterial DNA, are shown in Fig. 2. Correcting for both types of bacterial DNA contamination resulted in the complete removal of contaminating bacterial DNA from SMC samples generated using the micPCR/NGS methodology. In contrast, traditional PCR/NGS results still showed contaminating bacterial DNA present at 25 and 2.5 16S rRNA gene copies per organism, even after correction. This finding illustrates the higher accuracy of the micPCR/NGS methodology to quantify contaminating bacterial DNA from NEC samples and its requirement for the accurate subtraction of contaminating bacterial DNA from actual samples. In addition, the traditional PCR/NGS methodology failed to recover 25 16S rRNA gene copies of *M. catarrhalis* and hugely overestimated the abundance of *S. aureus* 16S rRNA gene copies in the SMC samples containing 2.5 16S rRNA gene copies per bacterium. Additionally, micPCR/NGS successfully detected all four bacterial species at 2,500, 250 and 25 16S rRNA gene molecules per bacterium, but failed to detect any of these species in triplicate experiments using 2.5 16S rRNA gene copies. We therefore estimated that the analytical sensitivity, indicated as the limit of detection (LOD), for micPCR/NGS is 25 16S rRNA gene molecules per OTU, whereas the LOD of traditional PCR/NGS was estimated as 250 16S rRNA gene copies per OTU.

In order to investigate the performance of the calibrated micPCR/NGS protocol using clinical samples, we selected four human skin swab samples that contained a low number of 16S rRNA gene copies (range: 64–604 16S rRNA gene copies/μL). The four samples, including an NEC, were processed in triplicate using micPCR/NGS and traditional PCR/NGS in parallel. Using micPCR/NGS, we obtained an average of 7,126 (±1,702) QC-passed sequence reads per sample, of which only 2 (±3) sequences per sample were identified and removed as chimeric sequences (Supplementary Table 8). Next, we were able to detect 3 to 13 OTUs in the samples, of which *Staphylococcus* and *Neisseria* species were commonly found and could be confirmed by bacterial culture. In addition, the micPCR/NGS method also detected the skin inhabitants *Streptococcus, Paracoccus, Enhydrobacter, Gemella, Sphingomonas, Alloprevotella, Propionibacterium, Brevundimonas, Roseomonas, Rothia, Granulicatella, Rhodococcus*, and *Flavobacteriaceae* species^8^,^9^. Importantly, correcting for contaminating bacterial DNA using the two-step strategy as described above removed 92% (range 89 − 96%) of the resultant OTUs found in the skin swab samples (Supplementary Table 9). Finally, we measured a high concordance between the total bacterial biomass when estimated indirectly using calibrated micPCR/NGS, without the correction for contaminating bacterial DNA, compared to the total 16S rRNA gene copies obtained when estimated directly using a 16S rRNA gene qPCR according to Yang *et al*.^10^, with an average of only a 1.03-fold difference (±0.34), demonstrating the accuracy of the micPCR/NGS method. In contrast to micPCR/NGS, traditional PCR/NGS was not able to generate any 16S rRNA gene amplicons from these low biomass skin swab samples as this method only generated non-specific, low molecular weight amplicons. These non-specific PCR products were most like generated by the relative excess of human DNA - compared to bacterial DNA - in the samples, as the PCR/NGS methodology successfully generated 16S rRNA gene amplicons in SMC samples (where only bacterial DNA and no human DNA was present). This result indicates that the LOD for the traditional PCR/NGS methodology is even higher for actual clinical samples than was previously estimated using our SMC samples.

## Discussion

In this report we show that a micelle PCR/NGS methodology (micPCR/NGS), in combination with a unique internal calibrator (IC) generates robust and accurate quantitative microbiota profiles. Micelle PCR is characterized by the clonal amplification of template DNA by the physical separation of reaction ingredients into a large number of reaction compartments. In contrast to traditional PCR, which is performed in a single reaction volume, the compartmentalization during micPCR allows accurate quantification of target sequences due to a lower susceptibility to variations in PCR amplicon efficiencies and amplification biases such as chimera formation, false priming and primer dimer formation^3^. Therefore, our calibrated micPCR/NGS method allows for the utilization of just a single correction factor, obtained from a single IC, to convert 16S rRNA gene sequence reads to 16S rRNA gene copies for each individual OTU present within a sample. In contrast, optimized traditional PCR amplification protocols^11^ or alternative spike-in approaches that employ traditional PCR amplification methods, such as SCML^12^, remain vulnerable to template-specific variations in PCR efficiencies that could easily result in error-derived microbiota profiles. However, it is true that the number of 16S rRNA gene copies produced using micPCR depends on the actual volume of individual micelles and that possible quantitation biases might be introduced due to differences in micelle sizes. This may be particularly relevant for the accurate quantification of low abundant taxa that are more vulnerable to the possible stochastic distribution of template DNA molecules into unevenly shaped micelles. However, the randomness of micelle size frequency distributions generated in independent experiments will tend to average out any possible quantification bias generated due to differences in the distribution of micelle sizes between independent experiments, as indicated by the results obtained using our synthetic microbial community (SMC).

The absolute quantification of OTUs using micPCR/NGS in combination with an internal calibrator improves the standardization of microbiota profiling results by removing the susceptibility to compositional effects. For example, traditional 16S rRNA gene sequencing methods require specific tools and methods that properly account for the statistical implications from the compositional structure of the data obtained^13^. But despite the growing interest and recent efforts to develop these sophisticated methods, the problems of spurious correlations in compositional data remain as yet unsolved^14^. In contrast, our results show that the use of the calibrated micPCR/NGS strategy greatly improves standardization of microbiota research without the reliance on (complex) compositional data analysis. However, these results remain vulnerable to contaminating bacterial DNA molecules derived from the sample processing environment. In order to correct for contaminating bacterial DNA, we eliminated OTUs that could not be reproducibly measured in triplicate experiments and subtracted 16S rRNA gene copies that were also quantified from negative extraction control (NEC) samples. The comparison of the micPCR/NGS and traditional PCR/NGS results showed that both methods possess a similar trueness when profiling the microbiota of synthetic microbial community (SMC) samples. However, the precision of the micPCR/NGS was much higher compared to the traditional PCR/NGS method. The low precision of the traditional PCR/NGS methodology resulted in unpredictable random errors within the microbiota profiles obtained, including those obtained from NEC samples. The increased random errors observed using traditional PCR/NGS makes the accurate subtraction of bacterial DNA contamination unreliable, whilst in contrast, the high precision of micPCR/NGS resulted in highly accurate quantification of (contaminating) 16S rRNA gene copies resulting in improved quantitative microbiota profiles that were free of contaminating bacterial DNA from environmental sources.

Using micPCR/NGS, we determined the quantitative microbiota profiles of low biomass skin swab samples. As expected, most OTUs (>89%) obtained from these clinical samples could not be reliably determined in triplicate, or the quantified number of 16S rRNA gene copies did not exceed the quantified number of the same 16S rRNA gene copies determined within NEC samples, and were removed accordingly. This finding stresses the importance of removing contaminating bacterial DNA from microbiota profiles obtained using low biomass samples. Additionally, traditional PCR/NGS was not able to generate any useful 16S rRNA gene sequencing data using the same clinical samples. This result is probably caused by other sample components than bacterial DNA, such as human DNA, that interferes with traditional PCR reactions (via inhibition or competition). This finding also illustrates the specific nature of PCR in micelles. Since all sample components (both 16S rRNA gene templates and non-templates) are limited to a single micelle, micPCR reactions are not affected by inhibiting or competing components and are still able to generate 16S rRNA gene amplicons successfully. However, it is important to note that possible effects of sample storage conditions^15^, cell lysis^16^, and primer specificity^17^ on the final results of these microbiota profiles still exist.

In summary, a combination of micPCR/NGS and an internal calibrator generates robust and accurate quantitative microbiota profiles. The high accuracy and low limit of detection of the calibrated micPCR/NGS, makes this method the preferential method to determine accurate and quantitative microbiota profiles for low biomass samples that are hampered by contaminating bacterial DNA. The general adoption of this approach by microbiota investigators will greatly improve the standardization of microbiota profiling results between individual experiments, laboratories and scientific publications.

## Methods

### Synthetic microbial community samples

The DNA used to create the SMC samples was extracted from four independently cultured bacterial strains; *Moraxella catarrhalis* (ATCC 25240), *Staphylococcus aureus* (ATCC 43300), *Haemophilus influenzae* (ATCC 10211), and *Clostridium perfringens* (ATCC 12915), using a phenol/bead-beating protocol combined with the AGOWA mag Mini DNA Isolation Kit (LGC) as described previously^6^. In addition, DNA from elution buffer BL (LGC) was extracted as a negative extraction control (NEC) sample at the same time to assess the composition of contaminating bacterial DNA in the experimental methodologies. In order to generate an equimolar mixture of 16S rRNA gene targets from the four bacterial DNA extracts, and to normalize the *Synechococcus* sp. (ATCC^®^ 27264D-5) DNA used as IC, the total dsDNA concentration from each DNA isolate was determined individually using the Quant-iT PicoGreen dsDNS assay Kit (Life Technologies) and normalized for genome sizes and 16S rRNA gene copy numbers based on bacterial whole-genome sequences that are publically available at the NCBI database. Next, a 10-fold dilution series of the equimolar SMC sample was made, ranging from 2,500 to 2.5 16S rRNA gene copies per organism. Prior to amplification by either micPCR or traditional PCR, 1,000, 100, 100, 50 and 50 16S rRNA gene copies of *Synechococcus* DNA was added as IC to the SMC DNA extracts containing 2,500, 250, 25, 2.5 16S rRNA gene copies per bacterial species and the NEC DNA extract, respectively.

### Skin swab samples

An acknowledged national ethics committee from the Netherlands (Medisch Ethische Toetsingscommissie Noord-Holland, http://www.metc.nl) approved the study protocol (M015–021) and all experiments were performed in accordance with the relevant guidelines and regulations. Skin swab samples were collected from patients with atopic dermatitis after written, informed consent was obtained from all subjects. Skin swab samples were collected using E-swabs (490CE, Copan) by gently rubbing the dry flocked swab over the dermatitis lesion (~2 cm^2^) for 10 seconds after which the entire sample was eluted upon contact with 1 mL liquid Amies preservation medium. All samples were cultured according to standard laboratory protocols performed in our laboratory and stored at −80 °C for subsequent 16S rRNA gene sequencing analysis. The routine culture methods included aerobic overnight culture at 35 degrees Celsius on CAP, TSASB and CLED agar plates after which Matrix-Assisted Laser Desorption Ionization Time-Of-Flight (MALDI-TOF) mass spectrometry was used for the identification of cultured bacterial species. For 16S rRNA gene sequencing analysis, DNA was extracted from the skin swab samples using the High Pure PCR Template Preparation Kit (Roche) according to the manufacturer’s instructions. In addition, DNA from Amies medium (490CE, Copan) was extracted as an NEC sample at the same time to allow contaminating bacterial DNA subtraction after NGS processing. Note that in this study we did not focus on DNA extraction efficiencies and the DNA extraction kit used may not be 100% efficient for determining microbial communities from skin swab samples. The total number of 16S rRNA gene copies within each DNA extract was measured using a 16S quantitative PCR (qPCR) according to Yang *et al*.^10^. For this, CT-values were related to a serial dilution of the previously calibrated and normalized SMC sample and ranged from a total of 100 to 10,000 16S rRNA gene copies per PCR reaction. Prior to amplification by either micPCR or traditional PCR, 50 16S rRNA gene copies of *Synechococcus* DNA was added as IC to the skin swab DNA extracts and the NEC DNA extract.

### Micelle PCR and traditional PCR amplification

16S rRNA gene amplicon library preparation using micPCR and traditional PCR was performed as previously published^3^, but with a slight modification. In this study, both amplification strategies were performed using modified 341F (5′-GACACTATAGCCTACGGGRSGCAGCAG-3′) and 806R (CACTATAGGGACTACNVGGGTWTCTAAT) primers that amplified the V3-V4 regions of 16S rRNA genes and which incorporated universal sequence tails at their 5′ ends to allow for a two-step amplification strategy. Also, both micPCR and traditional PCR were performed using the same PCR reagents (except for the oil phase used to generate the micelles) and PCR conditions following the Micelle PCR Amplification protocol as previously published^3^. Finally, both micPCR/NGS and the traditional PCR/NGS methodology utilized the same amplicon purification steps to synchronize experimental conditions.

### 16S rRNA gene sequencing and data analysis

Bidirectional sequencing of the 16S rRNA gene amplicon libraries was performed using the 454 Genome Sequencer (GS) Junior platform (Roche), with Fasta-formatted sequences being extracted from the GS Junior machine and further processed using the mothur v. 1.33.0 software package^18^. Primer sequences were trimmed and sequences that had an ambiguous base call (N) in the sequence or with lengths smaller than 400 were removed from the analysis. Unique sequences were then aligned against a customized reference alignment based on the SILVA reference alignment release 119 (available at: http://www.mothur.org/wiki/Silva_reference_alignment). The reference sequences were trimmed to only include the V3-V4 region of the 16S rRNA gene using the pcr.seqs command. Sequences that did not align to this region were culled from further analysis and the alignments were trimmed so that the sequences fully overlapped the same alignment coordinates. Potentially chimeric sequences were removed using Uchime, as implemented in mothur. The remaining sequences were classified using the classify.seqs command with the customized SILVA alignment release 119 as reference. Next, sequences were clustered into OTUs at 97% similarity using the default settings of the dist.seq and cluster commands respectively. The classify.otu algorithm was used to get a consensus taxonomy for each OTU. Finally, all SMC samples were rarefied to 1,000 sequences per sample and all skin swab samples were rarefied to 5,000 sequences per sample. The sequencing data that are connected to this article are uploaded to the Sequence Read Archive database with accession number SRP076831.

### Statistical analyses

The Kolmogorov-Smirnov test was used to check the normality of data distribution. Precision analyses were performed by calculating the coefficient of variation for each of the four OTUs obtained from the SMC samples. The paired Wilcoxon signed-rank test was used to compare the coefficients of variation obtained using micPCR/NGS and traditional PCR/NGS (SPSS version 23, IBM Corporation).

## Additional Information

**How to cite this article**: Boers, S. A. *et al*. Novel micelle PCR-based method for accurate, sensitive and quantitative microbiota profiling. *Sci. Rep.* **7**, 45536; doi: 10.1038/srep45536 (2017).

**Publisher's note:** Springer Nature remains neutral with regard to jurisdictional claims in published maps and institutional affiliations.

## Supplementary Material

Supplementary Data

## Figures and Tables

**Table 1:** Accuracy of 16S rRNA gene copy determination using synthetic microbial community (SMC) samples comparing the results of micPCR/NGS to traditional PCR/NGS.

OTU	Expected	micPCR/NGS					traditional PCR/NGS				
		Replicate 1	Replicate 2	Replicate 3	Trueness	Precision	Replicate 1	Replicate 2	Replicate 3	Trueness	Precision
*Clostridium*	2500	6,735	3,840	4,347	2.0	0.3	953	6,638	7,340	2.0	0.7
*Staphylococcus*	2500	4,776	3,147	4,875	1.7	0.2	403	3,793	4,075	1.1	0.7
*Haemophilus*	2500	4,082	3,133	2,611	1.3	0.2	370	2,483	2,509	0.7	0.7
*Moraxella*	2500	3,714	2,213	1,056	0.9	0.6	36	3,034	3,604	0.9	0.9
*Clostridium*	250	487	631	641	2.3	0.1	736	513	497	2.3	0.2
*Staphylococcus*	250	486	579	375	1.9	0.2	302	210	284	1.1	0.2
*Haemophilus*	250	238	225	183	0.9	0.1	281	226	261	1.0	0.1
*Moraxella*	250	225	363	214	1.1	0.3	240	188	231	0.9	0.1
*Clostridium*	25	28	31	52	1.5	0.3	119	29	36	2.4	0.8
*Staphylococcus*	25	57	47	52	2.1	0.1	15	27	50	1.2	0.6
*Haemophilus*	25	10	9	54	1.0	1.1	112	28	28	2.3	0.9
*Moraxella*	25	19	5	29	0.7	0.7	116	2	0	1.6	1.7
*Clostridium*	2,5	0	4	0	0.6	1.6	0	11	1	1.7	1.5
*Staphylococcus*	2,5	1	15	19	4.6	0.8	243	6	1	33.3	1.7
*Haemophilus*	2,5	1	0	9	1.3	1.6	0	0	10	1.3	1.7
*Moraxella*	2,5	1	3	0	0.6	1.1	0	7	0	1.0	1.6

The expected and measured values (Replicate 1–3) represent the number of 16S rRNA gene copies obtained for each individual bacterial species at four different input DNA concentrations (2,500, 250, 25 and 2.5 16S rRNA gene copies). The trueness shows the closeness of measurement results to the true (expected) value and was calculated by dividing the number of 16S rRNA gene copies measured (as an average of triplicate results) to the expected number of 16S rRNA gene copies present in the calibrated synthetic microbial community (SMC). The precision shows the coefficient of variation that was calculated by dividing the standard deviation obtained from triplicate results to the average number of 16S rRNA gene copies measured.
