# Supplementary material for: Novel micelle PCR-based method for accurate, sensitive and quantitative microbiota profiling
Source: Sci Rep. 2017 Apr 5;7:45536. doi: 10.1038/srep45536 (PMC5381217; doi:10.1038/srep45536)
Supplement: Supplementary Data [file srep45536-s1.pdf]

Novel micelle PCR method for accurate, sensitive and quantitative microbiota profiling

Stefan A. Boers, John P. Hays, Ruud Jansen

Supplementary Table 1. Sequence characteristics obtained from the synthetic microbial community (SMC) samples containing 2,500, 250, 25, 2.5 and 0 16S rRNA gene copies per bacterial species comparing the results of micPCR/NGS to PCR/NGS. All data represent average values obtained from triplicate experiments, of which the standard deviation is given in parentheses. The number of sequences that passed quality filtering includes sequence reads that contain the 16S rRNA gene primer sequences used (with a maximum of 2 differences to the primer sequence), were longer than 400 bases, did not contain any ambiguous base calls and could be aligned using the SILVA alignment release 119 as reference.

micPCR/NGS					
**Sample ID**	**SMC 2,500**	**SMC 250**	**SMC 25**	**SMC 2.5**	**SMC 0**
# raw sequences	4,944 (1,582)	5,823 (680)	5,976 (3,224)	5,517 (2,898)	8,340 (2,374)
# QC passed sequences	4,809 (1,505)	5,697 (614)	5,740 (3,299)	5,416 (2,852)	7,642 (1,564)
# chimeras removed	0 (0)	0 (1)	0 (1)	0 (0)	3 (5)
# normalized sequences	1,000	1,000	1,000	1,000	1,000
**traditional PCR/NGS**					
**Sample ID**	**SMC 2,500**	**SMC 250**	**SMC 25**	**SMC 2.5**	**SMC 0**
# raw sequences	2,112 (648)	3,383 (2,485)	2,951 (675)	8,304 (10,941)	2,923 (1,491)
# QC passed sequences	1,943 (615)	3,070 (2,307	2,243 (570)	2,876 (1,685)	2,576 (1,015)
# chimeras removed	142 (105)	208 (191)	101 (92)	58 (10)	66 (28)
# normalized sequences	1,000	1,000	1,000	1,000	1,000

Supplementary Table 2. Microbiota profiles obtained from the synthetic microbial community sample containing 2,500 16S rRNA gene copies per bacterial species comparing the results of micPCR/NGS to PCR/NGS. The microbiota profiles are expressed as a measure of 16S rRNA gene copies using the number of sequence reads of each individual OTU and the efficiency of the calibrated IC as a correction factor.

**Taxonomy (OTU#)**	**micPCR/NGS**						**traditional PCR/NGS**					
	**Replicate 1**		**Replicate 2**		**Replicate 3**		**Replicate 1**		**Replicate 2**		**Replicate 3**	
	**Reads**	**Copies**	**Reads**	**Copies**	**Reads**	**Copies**	**Reads**	**Copies**	**Reads**	**Copies**	**Reads**	**Copies**
Synechococcus (IC)	49	1000	75	1000	72	1000	362	1000	58	1000	53	1000
Clostridium (OTU272)	330	6735	288	3840	313	4347	345	953	385	6638	389	7340
Staphylococcus (OTU016)	234	4776	236	3147	351	4875	146	403	220	3793	216	4075
Haemophilus (OTU087)	200	4082	235	3133	188	2611	134	370	144	2483	133	2509
Moraxella (OTU046)	182	3714	166	2213	76	1056	13	36	176	3034	191	3604
Methanobrevibacter (OTU270)	-	-	-	-	-	-	-	-	10	172	-	-
Anaerococcus (OTU318)	-	-	-	-	-	-	-	-	-	-	4	75
Corynebacterium (OTU064)	-	-	-	-	-	-	-	-	-	-	4	75
Actinobacteria (OTU223)	3	61	-	-	-	-	-	-	-	-	-	-
Acinetobacter (OTU025)	2	41	-	-	-	-	-	-	-	-	-	-
Alicycliphilus (OTU325)	-	-	-	-	-	-	-	-	-	-	1	19

Supplementary Table 3. Microbiota profiles obtained from the synthetic microbial community sample containing 250 16S rRNA gene copies per bacterial species comparing the results of micPCR/NGS to PCR/NGS. The microbiota profiles are expressed as a measure of 16S rRNA gene copies using the number of sequence reads of each individual OTU and the efficiency of the calibrated IC as a correction factor.

Taxonomy (OTU#)	micPCR/NGS						traditional PCR/NGS					
	Replicate 1		Replicate 2		Replicate 3		Replicate 1		Replicate 2		Replicate 3	
	Reads	Copies	Reads	Copies	Reads	Copies	Reads	Copies	Reads	Copies	Reads	Copies
Synechococcus (IC)	63	100	52	100	63	100	58	100	78	100	67	100
Clostridium (OTU272)	307	487	328	631	404	641	427	736	400	513	333	497
Staphylococcus (OTU016)	306	486	301	579	236	375	175	302	164	210	190	284
Haemophilus (OTU087)	150	238	117	225	115	183	163	281	176	226	175	261
Moraxella (OTU046)	142	225	189	363	135	214	139	240	147	188	155	231
Lachnospiraceae (OTU0319)	-	-	-	-	-	-	-	-	26	33	-	-
Kocuria (OTU045)	-	-	-	-	-	-	3	5	-	-	16	24
Corynebacterium (OTU064)	-	-	-	-	12	19	-	-	-	-	1	1
Rhizobium (OTU224)	-	-	-	-	-	-	-	-	-	-	13	19
Corynebacterium (OTU022)	1	2	-	-	10	16	-	-	-	-	1	1
Propionibacterium (OTU226)	-	-	1	2	10	16	-	-	-	-	-	-
Acinetobacter (OTU220)	-	-	8	15	-	-	-	-	-	-	-	-
Spirosoma (OTU278)	-	-	-	-	9	14	-	-	-	-	-	-
Acetobacter (OTU017)	8	13	1	2	-	-	-	-	-	-	9	13
Actinobacteria (OTU223)	-	-	-	-	-	-	3	5	-	-	8	12
Enhydrobacter (OTU085)	6	10	-	-	-	-	-	-	-	-	-	-
Selenomonas (OTU275)	4	6	-	-	-	-	-	-	-	-	-	-
Prevotella (OTU276)	4	6	-	-	-	-	-	-	-	-	-	-
Haemophilus (OTU125)	-	-	-	-	4	6	-	-	-	-	-	-
Actinomyces (OTU093)	-	-	2	4	-	-	3	5	-	-	-	-
Pseudomonas (OTU396)	3	5	-	-	-	-	-	-	-	-	-	-
Rhodobacteraceae (OTU281)	-	-	-	-	-	-	-	-	-	-	3	4
Streptococcus (OTU019)	-	-	-	-	-	-	1	2	-	-	2	3
Bosea (OTU273)	2	3	-	-	-	-	-	-	-	-	-	-
Acidovorax (OTU228)	-	-	-	-	2	3	-	-	-	-	-	-
Anaerococcus (OTU063)	-	-	-	-	-	-	2	3	-	-	-	-
Mycoplasma (OTU127)	-	-	-	-	-	-	-	-	2	3	-	-
Psychrobacter (OTU254)	1	2	-	-	-	-	-	-	-	-	-	-
Paracoccus (OTU080)	-	-	1	2	-	-	-	-	-	-	-	-
Micrococcus (OTU023)	-	-	-	-	-	-	1	2	-	-	-	-
Janibacter (OTU176)	-	-	-	-	-	-	-	-	1	1	-	-
Brevundimonas (OTU101)	-	-	-	-	-	-	-	-	-	-	1	1

Supplementary Table 4. Microbiota profiles obtained from the synthetic microbial community sample containing 25 16S rRNA gene copies per bacterial species comparing the results of micPCR/NGS to PCR/NGS. The microbiota profiles are expressed as a measure of 16S rRNA gene copies using the number of sequence reads of each individual OTU and the efficiency of the calibrated IC as a correction factor.

**Taxonomy (OTU#)**	**micPCR/NGS**						**traditional PCR/NGS**					
	**Replicate 1**		**Replicate 2**		**Replicate 3**		**Replicate 1**		**Replicate 2**		**Replicate 3**	
	**Reads**	**Copies**	**Reads**	**Copies**	**Reads**	**Copies**	**Reads**	**Copies**	**Reads**	**Copies**	**Reads**	**Copies**
Synechococcus (IC)	459	100	365	100	269	100	193	100	383	100	254	100
Clostridium (OTU272)	130	28	114	31	140	52	229	119	112	29	91	36
Staphylococcus (OTU016)	261	57	173	47	139	52	28	15	104	27	126	50
Haemophilus (OTU087)	44	10	34	9	144	54	217	112	109	28	71	28
Moraxella (OTU046)	89	19	18	5	79	29	223	116	6	2	-	-
Enhydrobacter (OTU085)	9	2	22	6	-	-	1	1	-	-	103	41
Streptococcus (OTU019)	-	-	23	6	77	29	51	26	-	-	52	20
Streptococcus (OTU097)	-	-	-	-	-	-	-	-	84	22	-	-
Haemophilus (OTU125)	-	-	-	-	-	-	35	18	-	-	-	-
Acetobacter (OTU017)	-	-	5	1	24	9	8	4	63	16	8	3
Rhizobium (OTU224)	-	-	-	-	-	-	-	-	-	-	37	15
Paracoccus (OTU281)	-	-	9	2	-	-	-	-	-	-	33	13
Pseudomonas (OTU021)	-	-	45	12	-	-	-	-	-	-	23	9
Corynebacterium (OTU022)	-	-	7	2	33	12	-	-	20	5	10	4
Acinetobacter (OTU025)	-	-	37	10	-	-	-	-	-	-	3	1
Rhizobium (OTU262)	-	-	-	-	18	7	-	-	-	-	-	-
Pseudomonas (OTU396)	-	-	25	7	4	1	-	-	-	-	11	4
Flavobacteriaceae (OTU322)	-	-	-	-	-	-	13	7	-	-	-	-
Corynebacterium (OTU064)	-	-	21	6	4	1	-	-	-	-	17	7
Micrococcus (OTU023)	-	-	-	-	-	-	-	-	-	-	17	7
Propionibacterium (OTU226)	-	-	-	-	18	7	-	-	6	2	-	-
Pedobacter (OTU280)	-	-	22	6	-	-	-	-	-	-	-	-
Brevundimonas (OTU101)	-	-	-	-	-	-	-	-	-	-	12	5
Stenotrophomonas (OTU161)	-	-	16	4	-	-	-	-	-	-	-	-
Acinetobacter (OTU220)	8	2	-	-	11	4	-	-	-	-	-	-
Actinobacteria (OTU223)	-	-	12	3	9	3	-	-	-	-	10	4
Prevotella (OTU071)	-	-	-	-	-	-	-	-	-	-	10	4
Asticcacaulis (OTU283)	-	-	-	-	10	4	-	-	-	-	-	-
Veillonellaceae (OTU284)	-	-	-	-	10	4	-	-	-	-	-	-
Alphaproteobacteria (OTU389)	-	-	-	-	-	-	-	-	-	-	8	3
Rhodobacteraceae (OTU397)	-	-	-	-	-	-	-	-	-	-	8	3
Paracoccus (OTU080)	-	-	9	2	-	-	-	-	-	-	7	3
Dermacoccus (OTU321)	-	-	-	-	-	-	2	1	-	-	7	3
Corynebacterium (OTU118)	-	-	-	-	-	-	-	-	-	-	7	3

Prevotella (OTU331)	-	-	-	-	-	-	-	-	-	-	7	3
Veillonella (OTU092)	-	-	-	-	-	-	-	-	-	-	7	3
Janibacter (OTU176)	-	-	-	-	-	-	-	-	-	-	6	2
Acinetobacter (OTU279)	-	-	8	2	-	-	-	-	-	-	-	-
Acinetobacter (OTU086)	-	-	-	-	-	-	-	-	-	-	5	2
Lautropia (OTU282)	-	-	-	-	5	2	-	-	-	-	-	-
Brevibacterium (OTU357)	-	-	6	2	-	-	-	-	-	-	-	-
Sporolactobacillus (OTU380)	-	-	6	2	-	-	-	-	-	-	-	-
Atopobium (OTU329)	-	-	-	-	-	-	-	-	-	-	4	2
Hydrogenophaga (OTU237)	-	-	-	-	-	-	-	-	-	-	4	2
Actinomyces (OTU093)	-	-	5	1	-	-	-	-	-	-	-	-
Enterobacter (OTU031)	-	-	-	-	-	-	-	-	-	-	3	1
Finegoldia (OTU032)	-	-	4	1	-	-	-	-	-	-	-	-
Anaerococcus (OTU002)	-	-	3	1	-	-	-	-	-	-	-	-
Blastocatella (OTU381)	-	-	3	1	-	-	-	-	-	-	-	-
Idiomarina (OTU388)	-	-	-	-	-	-	-	-	-	-	2	1
Nocardioides (OTU246)	-	-	-	-	-	-	-	-	-	-	2	1
Simonsiella (OTU115)	-	-	-	-	2	1	-	-	-	-	-	-
Chryseobacterium (OTU382)	-	-	2	1	-	-	-	-	-	-	-	-

Supplementary Table 5. Microbiota profiles obtained from the synthetic microbial community sample containing 2.5 16S rRNA gene copies per bacterial species comparing the results of micPCR/NGS to PCR/NGS. The microbiota profiles are expressed as a measure of 16S rRNA gene copies using the number of sequence reads of each individual OTU and the efficiency of the calibrated IC as a correction factor.

Taxonomy (OTU#)	micPCR/NGS						traditional PCR/NGS					
	Replicate 1		Replicate 2		Replicate 3		Replicate 1		Replicate 2		Replicate 3	
	Reads	Copies	Reads	Copies	Reads	Copies	Reads	Copies	Reads	Copies	Reads	Copies
Synechococcus (IC)	414	50	334	50	447	50	82	50	321	50	360	50
Clostridium (OTU272)	-	-	30	4	-	-	-	-	71	11	10	1
Staphylococcus (OTU016)	5	1	103	15	167	19	398	243	39	6	5	1
Haemophilus (OTU087)	5	1	-	-	83	9	-	-	-	-	69	10
Moraxella (OTU046)	10	1	23	3	-	-	-	-	47	7	-	-
Shuttleworthia (OTU336)	-	-	-	-	-	-	194	118	9	1	-	-
Actinomyces (OTU093)	21	3	27	4	52	6	111	68	8	1	45	6
Oxalobacteraceae (OTU296)	-	-	-	-	-	-	53	32	-	-	-	-
Acinetobacter (OTU084)	241	29	-	-	-	-	-	-	-	-	4	1
Corynebacterium (OTU064)	8	1	179	27	49	5	14	9	84	13	-	-
Actinobacteria (OTU223)	51	6	-	-	-	-	30	18	30	5	54	8
Streptococcus (OTU019)	24	3	58	9	84	9	28	17	42	7	90	13
Paracoccus (OTU080)	-	-	66	10	-	-	-	-	-	-	-	-
Corynebacterium (OTU290)	-	-	-	-	-	-	15	9	-	-	-	-
Acinetobacter (OTU025)	-	-	17	3	-	-	-	-	47	7	-	-
Enterobacteriaceae (OTU31)	-	-	-	-	-	-	12	7	-	-	-	-
Sphingomonas (OTU100)	-	-	-	-	-	-	-	-	-	-	52	7
Corynebacterium (OTU022)	13	2	47	7	40	4	10	6	21	3	31	4
Bifidobacterium (OTU341)	-	-	-	-	-	-	-	-	43	7	-	-
Aliidiomarina (OTU333)	-	-	-	-	-	-	8	5	-	-	-	-
Rothia (OTU170)	-	-	-	-	-	-	-	-	19	3	35	5
Sphingopyxis (OTU287)	39	5	-	-	-	-	-	-	-	-	-	-
Acetobacter (OTU017)	-	-	18	3	-	-	-	-	29	5	-	-
Bacillus (OTU293)	-	-	30	4	-	-	-	-	-	-	-	-
Micrococcaceae (OTU299)	-	-	29	4	-	-	-	-	-	-	-	-
Veillonella (OTU092)	35	4	-	-	-	-	-	-	-	-	4	1
Haemophilus (OTU125)	-	-	-	-	37	4	-	-	7	1	-	-
Lactobacillus (OTU351)	-	-	-	-	-	-	-	-	-	-	29	4
Anaerococcus (OTU063)	-	-	-	-	-	-	-	-	14	2	28	4
Alphaproteobacteria (OTU392)	-	-	-	-	-	-	6	4	-	-	-	-
Pseudomonadaceae (OTU349)	-	-	-	-	-	-	-	-	-	-	26	4
Dialister (OTU344)	-	-	-	-	-	-	-	-	22	3	-	-
Pelagibacterium (OTU339)	-	-	-	-	-	-	-	-	21	3	-	-

Rothia (OTU044)	-	-	-	-	-	-	5	3	-	-	19	3
Micrococcaceae (OTU289)	25	3	-	-	-	-	-	-	-	-	-	-
Veillonella (OTU160)	25	3	-	-	-	-	-	-	-	-	-	-
Kocuria (OTU045)	-	-	-	-	26	3	-	-	-	-	-	-
Peptoniphilus (OTU004)	-	-	19	3	-	-	-	-	-	-	-	-
Paenalcaligenes (OTU285)	16	2	-	-	-	-	-	-	17	3	-	-
Nocardioides (OTU225)	-	-	-	-	-	-	-	-	17	3	-	-
Micrococcales (OTU288)	20	2	-	-	-	-	-	-	-	-	-	-
Escherichia-Shigella (OTU041)	-	-	-	-	-	-	-	-	-	-	15	2
Dechloromonas (OTU347)	-	-	-	-	-	-	-	-	-	-	15	2
Aquabacterium (OTU091)	-	-	-	-	-	-	-	-	13	2	-	-
Pusillimonas (OTU286)	14	2	-	-	-	-	-	-	8	1	14	2
Actinomyces (OTU383)	-	-	-	-	14	2	-	-	-	-	-	-
Pseudomonas (OTU348)	-	-	-	-	-	-	-	-	-	-	11	2
Pseudomonas (OTU396)	-	-	-	-	-	-	-	-	9	1	10	1
TM7 (OTU345)	-	-	-	-	-	-	-	-	9	1	-	-
Streptococcus (OTU082)	-	-	-	-	-	-	-	-	-	-	10	1
Enhydrobacter (OTU085)	-	-	9	1	-	-	-	-	-	-	-	-
Jeotgalicoccus (OTU140)	-	-	-	-	-	-	-	-	-	-	9	1
Rhodobacteraceae (OTU340)	-	-	-	-	-	-	-	-	8	1	-	-
Marinomonas (OTU337)	-	-	-	-	-	-	-	-	8	1	-	-
Paracoccus (OTU281)	7	1	-	-	-	-	2	1	-	-	-	-
Neisseria (OTU334)	10	1	-	-	-	-	1	1	-	-	-	-
Atopostipes (OTU210)	-	-	8	1	-	-	-	-	-	-	-	-
Olsenella (OTU310)	-	-	-	-	-	-	1	1	7	1	-	-
Chryseobacterium (OTU353)	-	-	-	-	-	-	-	-	-	-	7	1
SM1A02 (OTU355)	-	-	-	-	-	-	-	-	-	-	7	1
Amaricoccus (OTU350)	-	-	-	-	-	-	-	-	-	-	6	1
Mycoplasma (OTU127)	-	-	-	-	-	-	-	-	-	-	6	1
Rhizobiales (OTU338)	-	-	-	-	-	-	-	-	5	1	5	1
Dolosigranulum (OTU343)	-	-	-	-	-	-	-	-	5	1	-	-
Neisseriaceae (OTU115)	6	1	-	-	-	-	-	-	-	-	-	-
Luteimonas (OTU297)	-	-	-	-	-	-	-	-	-	-	5	1
Propionibacterium (OTU226)	-	-	-	-	-	-	1	1	4	1	-	-
Neisseria (OTU143)	-	-	-	-	-	-	-	-	4	1	-	-

Supplementary Table 6. Microbiota profiles obtained from the negative extraction control (NEC) sample using micPCR/NGS. The microbiota profiles are expressed as a measure of 16S rRNA gene copies using the number of sequence reads of each individual OTU and the efficiency of the calibrated IC as a correction factor. Only the OTUs that are found in all three independent measurements is presented.

**Taxonomy (OTU#)**	**Replicate 1**		**Replicate 2**		**Replicate 3**		**Average number of 16S copies + 3*STD**
	**Reads**	**Copies**	**Reads**	**Copies**	**Reads**	**Copies**	
Synechococcus (IC)	342	50,0	286	50,0	415	50,0	50
Streptococcus (OTU019)	65	9,5	146	25,5	7	0,8	12
Staphylococcus (OTU016)	73	10,7	83	14,5	48	5,8	10
Pseudomonas (OTU021)	18	2,6	66	11,5	58	7,0	7
Corynebacterium (OTU022)	14	2,0	58	10,1	41	4,9	6
Corynebacterium (OTU064)	23	3,4	20	3,5	57	6,9	5
Actinomyces (OTU093)	2	0,3	4	0,7	16	1,9	1
Propionibacterium (OTU226)	8	1,2	2	0,3	2	0,2	1

Supplementary Table 7. Microbiota profiles obtained from the negative extraction control (NEC) sample using PCR/NGS. The microbiota profiles are expressed as a measure of 16S rRNA gene copies using the number of sequence reads of each individual OTU and the efficiency of the calibrated IC as a correction factor. Only the OTUs that are found in all three independent measurements is presented.

Taxonomy (OTU#)	Replicate 1		Replicate 2		Replicate 3		Average number of 16S copies + 3*STD
	Reads	Copies	Reads	Copies	Reads	Copies	
Synechococcus (IC)	331	50,0	377	50,0	293	50,0	50
Atopostipes (OTU210)	7	1,1	21	2,8	64	10,9	21
Janthinobacterium (OTU296)	71	10,7	70	9,3	68	11,6	14
Streptococcus (OTU019)	27	4,1	60	8,0	39	6,7	12
Haemophilus (OTU125)	5	0,8	4	0,5	29	4,9	10
Staphylococcus (OTU016)	26	3,9	48	6,4	22	3,8	9
Actinobacteria (OTU223)	12	1,8	14	1,9	25	4,3	7
Corynebacterium (OTU022)	27	4,1	13	1,7	15	2,6	6

Supplementary Table 8. Sequence characteristics obtained from four skin swab samples and the corresponding negative extraction control using micPCR/NGS. All data represent average values obtained from triplicate experiments, of which the standard deviation is given in parentheses. The number of sequences that passed quality filtering includes sequence reads that contain the 16S rRNA gene primer sequences used (with a maximum of 2 differences to the primer sequence), were longer than 400 bases, did not contain any ambiguous base calls and could be aligned using the SILVA alignment release 119 as reference.

micPCR/NGS					
**Sample ID**	**Skin swab 1**	**Skin swab 2**	**Skin swab 3**	**Skin swab 4**	**NEC**
# raw sequences	8,996 (1,490)	8,613 (439)	5,638 (203)	5,943 (627)	7,323 (2,145)
# QC passed sequences	8,827 (1,435)	8,343 (451)	5,496 (174)	5,824 (609)	7,139 (2,124)
# chimeras removed	0 (1)	0 (1)	2 (3)	0 (1)	7 (3)
# normalized sequences	5,000	5,000	5,000	5,000	5,000

Supplementary Table 9. Microbiota profiles obtained from four skin swab samples comparing the results of micPCR/NGS to conventional culture. The microbiota profiles obtained using micPCR/NGS are expressed as a measure of 16S rRNA gene copies using the number of sequence reads of each individual OTU and the efficiency of the calibrated IC as a correction factor. OTUs found in all three independent measurements per sample are included and the average of the triplicate results is presented before and after correction for contaminated 16S rRNA gene copies found in all three independent measurements of the negative extraction control (NEC) sample.

**Skin swab sample 1**				
**Taxonomy (OTU#)**	**Sample (range)**	**NEC**	**Sample - NEC**	**Culture results**
Staphylococcus (OTU016)	294 (200-378)	24	270	*S. aureus*
Enhydrobacter (OTU085)	8 (4-11)	-	8	-
Paracoccus (OTU080)	8 (5-11)	-	8	-
Corynebacterium (OTU064)	7 (2-13)	12	-	-
Sphingomonas (OTU096)	6 (2-9)	-	6	-
Brevundimonas (OTU101)	4 (2-6)	-	4	-
Roseomonas (OTU0164)	3 (1-6)	-	3	-
Streptococcus (OTU019)	3 (1-5)	3	-	-
Corynebacterium (OTU022)	1 (1-2)	6	-	-
**Skin swab sample 2**				
**Taxonomy (OTU#)**	**Sample (range)**	**NEC**	**Sample - NEC**	**Culture results**
Streptococcus (OTU019)	45 (33-56)	3	42	-
Staphylococcus (OTU016)	24 (10-39)	24	-	*S. warneri, S. haemolyticus*
Neisseria (OTU334)	9 (3-16)	-	9	*Neisseria cinerea*
Gemella (OTU302)	8 (2-16)	-	8	-
Enhydrobacter (OTU085)	7 (1-13)	-	7	-
Paracoccus (OTU080)	7 (4-9)	-	7	-
Propionibacterium (OTU226)	6 (1-14)	1	5	-
Actinobacteria (OTU223)	6 (2-12)	10	-	-
Alloprevotella (OTU014)	6 (1-13)	-	6	-
Paracoccus (OTU281)	5 (4-6)	-	5	-
Haemophilus (OTU125)	4 (3-6)	8	-	-
Corynebacterium (OTU022)	3 (2-5)	6	-	-
Granulicatella (OTU175)	2 (1-3)	-	2	-
Roseomonas (OTU164)	2 (1-3)	-	2	-
Rhodococcus (OTU421)	2 (1-4)	-	2	-
Flavobacteriaceae (OTU428)	1 (1-3)	-	1	-
Granulicatella (OTU311)	1 (1-2)	-	1	-
**Skin swab sample 3**				
**Taxonomy (OTU#)**	**Sample (range)**	**NEC**	**Sample - NEC**	**Culture results**
Corynebacterium (OTU064)	11 (4-21)	12	-	-
Staphylococcus (OTU016)	7 (3-11)	24	-	*S. haemolyticus, S. epidermidis*
Streptococcus (OTU019)	7 (3-12)	3	4	-

Actinobacteria (OTU223)	5 (1-12)	10	-	-
Corynebacterium (OTU022)	3 (2-5)	6	-	-
Rothia (OTU044)	3 (2-4)	-	3	-
Paracoccus (OTU281)	1 (1)	-	1	-
**Skin swab sample 4**				
**Taxonomy (OTU#)**	**Sample (range)**	**NEC**	**Sample - NEC**	**Culture results**
Staphylococcus (OTU016)	49 (26-68)	24	25	*S. aureus, S. capitis*
Paracoccus (OTU080)	27 (19-34)	-	27	-
Streptococcus (OTU019)	26 (14-48)	3	23	-
Corynebacterium (OTU064)	11 (3-23)	12	-	-
Neisseria (OTU334)	4 (2-5)	-	4	-
Paracoccus (OTU281)	2 (1-3)	-	2	-
Corynebacterium (OTU022)	2 (1-2)	6	-	-
Actinobacteria (OTU223)	1 (1)	10	-	-
